# Diagnostic and vaccine potential of Zika virus envelope protein (E) derivates produced in bacterial and insect cells

**DOI:** 10.3389/fimmu.2023.1071041

**Published:** 2023-03-16

**Authors:** Victória Alves Santos Lunardelli, Bianca da Silva Almeida, Juliana de Souza Apostolico, Thais Rezende, Marcio Massao Yamamoto, Samuel Santos Pereira, Maria Fernanda Campagnari Bueno, Lennon Ramos Pereira, Karina Inacio Carvalho, Renata Dezengrini Slhessarenko, Luis Carlos de Souza Ferreira, Silvia Beatriz Boscardin, Daniela Santoro Rosa

**Affiliations:** ^1^ Departmento de Microbiologia, Imunologia e Parasitologia, Universidade Federal de São Paulo- Escola Paulista de Medicina (UNIFESP/EPM), São Paulo, Brazil; ^2^ Departmento de Parasitologia, Instituto de Ciências Biomédicas, Universidade de São Paulo (USP), São Paulo, Brazil; ^3^ Departmento de Microbiologia, Instituto de Ciências Biomédicas, Universidade de São Paulo (USP), São Paulo, Brazil; ^4^ Hospital Israelita Albert Einstein, São Paulo, Brazil; ^5^ Case Comprehensive Cancer Center, Case Western Reserve University, Cleveland, OH, United States; ^6^ Laboratório de Virologia, Universidade Federal do Mato Grosso, Cuiabá, Brazil; ^7^ Plataforma Científica Pasteur- Universidade de São Paulo, São Paulo, Brazil; ^8^ Instituto Nacional de Ciência e Tecnologia (INCT) de Investigação em Imunologia (iii), São Paulo, Brazil

**Keywords:** subunit vaccines, Zika virus, envelope protein (E), recombinant protein, envelope domain

## Abstract

**Introduction:**

In the present study we evaluated the features of different recombinant forms of Zika virus (ZIKV) proteins produced in either bacterial (*Eschericha coli*) or insect cells (*Drosophila melanogaster*). The ZIKV-envelope glycoprotein (E_ZIKV_) is responsible for virus entry into host cells, is the main target of neutralizing antibodies and has been used as a target antigen either for serological tests or for the development of subunit vaccines. The E_ZIKV_ is composed of three structural and functional domains (EDI, EDII, and EDIII), which share extensive sequence conservation with the corresponding counterparts expressed by other flaviviruses, particularly the different dengue virus (DENV) subtypes.

**Methods:**

In this study, we carried out a systematic comparison of the antigenicity and immunogenicity of recombinant EZIKV, EDI/IIZIKV and EDIIIZIKV produced in E. coli BL21 and Drosophila S2 cells. For the antigenicity analysis we collected 88 serum samples from ZIKV-infected participants and 57 serum samples from DENV-infected. For immunogenicity, C57BL/6 mice were immunized with two doses of EZIKV, EDI/IIZIKV and EDIIIZIKV produced in E. coli BL21 and Drosophila S2 cells to evaluate humoral and cellular immune response. In addition, AG129 mice were immunized with EZIKV and then challenge with ZIKV.

**Results:**

Testing of samples collected from ZIKV-infected and DENV-infected participants demonstrated that the EZIKV and EDIIIZIKV produced in BL21 cells presented better sensitivity and specificity compared to proteins produced in S2 cells. In vivo analyses were carried out with C57BL/6 mice and the results indicated that, despite similar immunogenicity, antigens produced in S2 cells, particularly EZIKV and EDIIIZIKV, induced higher ZIKV-neutralizing antibody levels in vaccinated mice. In addition, immunization with EZIKV expressed in S2 cells delayed the onset of symptoms and increased survival rates in immunocompromised mice. All recombinant antigens, either produced in bacteria or insect cells, induced antigen-specific CD4+ and CD8+ T cell responses.

**Conclusion:**

In conclusion, the present study highlights the differences in antigenicity and immunogenicity of recombinant ZIKV antigens produced in two heterologous protein expression systems.

## Introduction

Zika virus (ZIKV) attracted world attention after recent outbreaks, leading the World Health Organization (WHO) to declare Zika fever as a public health emergency of international concern. To date, more than 80 countries have already reported mosquito-borne ZIKV infection (1). ZIKV is a flavivirus, transmitted mainly by the bite of female *Aedes* mosquitoes, but sexual and vertical transmissions have also been reported (2, 3). Most ZIKV infections are mild or asymptomatic; however, ZIKV recently re-emerged associated with neurological disorders such as Congenital Zika Syndrome (CZS) (4–7) and Guillain-Barré Syndrome (GBS) (8, 9).

The ZIKV genome encodes three structural proteins (premembrane [prM], envelope [E], and capsid [C]), and seven nonstructural proteins (NS1, NS2A, NS2B, NS3, NS4A, NS4B, and NS5) (10). Nonstructural proteins are essential for viral replication and polyprotein processing, while structural proteins play an important role in virus particle morphogenesis. The E protein is responsible for viral entry and membrane fusion and consists of three ectodomains: EDI (central domain), EDII (dimerization domain and EDIII (receptor binding domain) (11). The ZIKV E protein is closely related to the envelope of dengue virus (DENV1-4) – ranging from 54 to 57.8% of amino acid conservation (12), which results in a cross-reactive antibody response between both viruses (13, 14). Antibodies generated against the EDI and EDII subdomains are more likely to cross-react between ZIKV and DENV, as compared to EDIII (15–17). ZIKV diagnosis is based on the viral RNA detection by RT-PCR or antibody-detection tests such as ELISA and indirect immunofluorescence. The plaque reduction neutralization test (PRNT) is used as the gold standard to resolve inconclusive results and confirm cross-reactive antibody results (18). Although IgM antibodies against ZIKV usually appear five days after the onset of the symptoms and remain detectable for a short period, anti-ZIKV IgG levels last for at least six months after infection (19). For this reason, detection of circulating ZIKV IgG antibodies continues to be widely used to identify prior ZIKV exposure. However, the high cross-reactivity of ZIKV-antibodies with other flaviviruses hinders the accurate diagnosis of both infections (20, 21).

Despite substantial efforts, we still do not have a licensed ZIKV effective vaccine (22). So far, several candidates have already been tested into phase I and II clinical trials, including live attenuated, whole inactivated, viral vectored, DNA and mRNA-based vaccines (23). The ZIKV E protein is highly immunogenic and induces potent neutralizing antibodies response that confer protection in animal models (14, 24–26). Monoclonal antibodies (mAbs) targeting the E region inhibits ZIKV infection (15, 26–28), as well as passive transfer of E-specific mAbs reduces viral infection (24). Moreover, EDIII confers the highest neutralizing antibodies (15). Therefore, the envelope region is the preferred target for designing a subunit vaccine against ZIKV and for serological tests (29). In both scenarios, the expression system used to produce the E protein may affect the ability to induce specific and protective immune responses (30–33). The prokaryotic system is the most commonly used for protein production, due to its high protein yield (34). However, the eukaryote system is used when a proper protein folding is required, especially for proteins that are normally produced in eukaryotic cells such as it is the case of E_ZIKV_ protein (35). Indeed, antibodies against E protein dimmer (quaternary epitopes) are responsible for high levels of neutralization (36). For this reason, the choice of the expression system for vaccine production is of utmost importance and can affect the protective efficacy of vaccine formulations based on recombinant proteins.

Here, we compared the immune responses against different ZIKV-envelope proteins produced in two expression systems: the *Escherichia coli* BL21 and *Drosophila melanogaster* S2 cells and show differences in the antigenicity and immunogenicity of the produced ZIKV proteins. This information can contribute for further diagnostic or prophylactic use of ZIKV antigens.

## Materials and methods

### E_ZIKV_, EDI/II_ZIKV_ and EDIII_ZIKV_ sequences

The ZIKV envelope (E_ZIKV_) sequence (amino acids 291-690 of the ZIKV polyprotein) was generated as previously described (37). The sequences were codon optimized for prokaryote or eykaryote expression (GenScript, NJ) and cloned into pET21a plasmid using *NheI* and *XhoI* restriction sites (named as pET21a-E_ZIKV_) and in pVAX (pVAX-E_ZIKV_) vector using *HindIII*/*XhoI*. Then, the ectodomains EDI/II_ZIKV_ (aa 291-600) and EDIII_ZIKV_ (aa 601-690) sequences were amplified by PCR with specific primers (**Supplementary Table 1**) using Phusion High Fidelity DNA Polymerase (New England Biolabs) according to the manufacturer’s instructions. The PCR products were cloned into the pJET1.2/blunt vector (Thermo Scientific) and digested with restriction enzymes *NheI* and *XhoI* (New England Biolabs) for bacteria expression and *NcoI* and *XhoI* for S2 expression. The digested fragment was purified using PureLink Quick Plasmid DNA kit (Invitrogen) and cloned in frame with the open reading frames of pET21a or pMT/BiP/V5-HisB vector using T4 DNA ligase enzyme (New England Biolabs). Plasmids were sequenced and transformed into DH5α bacteria for DNA purification using the EndoFree Plasmid Maxi Kit (Qiagen) according to the manufacturer’s instructions.

### E_ZIKV_, EDI/II_ZIKV_ and EDIII_ZIKV_ protein expression and purification

For prokaryote production, E_ZIKV_, EDI/II_ZIKV_ and EDIII_ZIKV_ recombinant proteins were produced as previously described (37). Briefly, the *E.coli* BL21 (DE3) RIL strain harboring the plasmid pET21a-E_ZIKV_, pET21a-EDI/II_ZIKV_ or pET21a-EDIII_ZIKV_ was cultivated in LB medium containing ampicillin (100 μg/ml) plus chloramphenicol (25 μg/ml). When the culture reached OD_600nm_ between 0.6-0.8, IPTG 0.01mM (Sigma) was added for 4 hours at 37°C and 200 rpm. After harvested (15 minutes, 4°C and 5,900 x g), the bacterial pellet was suspended in Buffer A (Tris-HCl 100 mM, NaCl 500 mM, glycerol 15%, pH 8) and lysed in a high-pressure system (600 bar, 10 minutes, 4°C) (APLAB-10, ARTEPEÇAS-Brazil). The inclusion bodies were then slowly dripped (250μL/mL) into Buffer A supplemented with 8M urea overnight under slow and constant stirring at 4°C. The supernatant with soluble protein was refolded in Buffer A supplemented with 20mM of 2-mercaptoethanol. The E_ZIKV_, EDI/II_ZIKV_ and EDIII_ZIKV_ soluble recombinant proteins were purified using a nickel affinity chromatography Ni-NTA-Agarose columns (ThermoFisher Scientific) according to the manufacturer’s instructions. The envelope proteins from DENV (E_DENV2_ and EDIII_DENV2_) were produced as previously described (38).

For S2 Drosophila *melanogaster* expression system, 5x10^5^ S2 cells/mL were grown in Schneider’s Drosophila complete medium (SDM) (Gibco) supplemented with 5% fetal bovine serum (FBS) (Gibco), 0.5x penicillin-streptomycin (Gibco) in a 6 well plate (Costar). After 24h, transfection was performed with 5μg of produced vector (pMT-E_ZIKV_, pMT-EDI/II_ZIKV_ or pMT-EDIII_ZIKV_) plus 0.25μg of pCoBlast (ThermoFisher Scientific) and 11.25μg of polyethyleneimine (PEI) in a total volume of 100μL of 150mM NaCl solution. Seventy-two hours after transfection, the medium was changed to complete SDM containing 15μg/ml blasticidin (ThermoFisher Scientific). After 2 to 4 weeks of selection, resistant cell cultures were expanded and evaluated for protein expression. To this end, 700μM of CuSO_4_ were add to the culture. After seven days of incubation, the expression of proteins in the culture supernatant was evaluated. For large-scale production, stable S2 cells expressing E_ZIKV_, EDI/II_ZIKV_ or EDIII_ZIKV_ were incubated at 28°C in 100mL of SDM containing 15μg of blasticidin until reaching a concentration of 10^7^ cells/mL. The medium was then replaced with SDM medium without FBS containing 700μM CuSO_4_. After seven days, the cell supernatant was collected and 1mM PMSF was added. Next, the supernatant was centrifuged at 3,000g for 5 minutes and filtered through 0.22μM membranes. The E_ZIKV_, EDI/II_ZIKV_ and EDIII_ZIKV_ soluble recombinant proteins were purified using a nickel affinity chromatography Ni-NTA-Agarose columns (ThermoFisher Scientific) according to the manufacturer’s instructions.

### Collection of human samples

Serum samples from ZIKV-infected individuals (n=88) were collected in 2016 and 2017 after a ZIKV outbreak in Brazil. All samples were tested for DENV, ZIKV and Chikungunya virus infection by IIFT Arboviral Fever Mosaic 2 IgG/IgM (EUROIMMUN). Serum samples from Brazilian DENV-infected individuals (n=57) were collected in 2012 and 2013 before ZIKV outbreak. Serum from non-infected (DENV^-^ZIKV^-^) individuals (n=18) were used as negative control. All procedures were approved by the Ethics Committee (protocol CAAE 68688117.0.0000.5505).

### Mice, immunization and challenge

Six- to eight-weeks-old female C57Bl/6 mice were bred at Centro de Desenvolvimento de Modelos Experimentais para Medicina e Biologia (CEDEME) – UNIFESP. All mice were housed in a temperature-controlled, light-cycled facility at Division of Immunology – UNIFESP. All experiments using mice in this study were approved by the UNIFESP Institutional Animal Care and Use Committee (IACUC) under protocol number #2020100418 and were in accordance with the recommendations of the Federal Law 11.794 (2008), the Guide for the Care and Use of Laboratory Animals of the Brazilian National Council of Animal Experimentation (CONCEA) and the ARRIVE guidelines (https://arriveguidelines.org). For immunization, C57BL/6 mice received two doses, at 2-week intervals, with equimolar amounts of prokaryote or eukaryote E_ZIKV_ (10 μg), EDI/II_ZIKV_ (7.78 μg) or EDIII_ZIKV_ (2.44 μg) in the presence of the adjuvant AddaVax (1:1 v/v; *In vivo*gen) in a total volume of 100 μL delivered subcutaneously (s.c.) – at the base of the tail. Blood was collected by submandibular vein fourteen days after each dose and mice were euthanized two weeks after the last dose. For *in vivo* neutralization, serum from C57Bl/6 mice immunized with E_ZIKV_ or only AddaVax adjuvant (1:10) were incubated for 1h with 100 PFU of ZIKV isolate (ZIKV^BR^), described by Cugola et al. (39). After this period, AG129 mice received the serum-virus mixture into footpads. Weight, symptoms and survival rate were monitored until 15 days post-infection. For challenge AG129 mice were immunized with prokaryote or eukaryote E_ZIKV_ in the same conditions as described above. Fifteen days after boost, mice were challenge with 100 PFU of ZIKV into footpads. Weight, symptoms, and survival rate were monitored until 18 days post-infection. The animals were submitted to euthanasia if we detected a loss of 20% of the initial weight and/or worsening of symptoms.

### Western blotting

Purified recombinant E_ZIKV_, EDI/II_ZIKV_ and EDIII_ZIKV_ proteins (500ng) were submitted to 15% SDS-PAGE gel under reducing conditions and transferred to nitrocellulose membranes (Hybond-C extra nitrocellulose – GE Healthcare). Next, membranes were blocked overnight at 4°C with PBS containing Tween 20 (PBST) (0.05% v/v), non-fat milk (5% w/v) and BSA (2.5% w/v). After each subsequent step the membranes were washed 3 times with PBST. Then, the membranes were incubated with anti-his 6x tag (1:5000 ThermoFisher Scientific) or serum from ZIKV-infected patient (1:500) at room temperature (rt) for 2 hours (h). After, the membranes were incubated with horseradish peroxidase-labeled goat anti-mouse IgG (1:5,000; KPL) or alkaline phosphatase goat anti-human IgG (1:16,000; KPL) at rt for 1h. The reaction was developed with a chemiluminescence kit (ECL, GE Healthcare) or using the commercial kit NBT/BCIP (Invitrogen) according to manufacturer’s instructions and analyzed with Alliance 4.7 software (Uvitec; Cambridge).

### Measurement of ZIKV-antibodies against different envelope proteins by ELISA

ELISA plates (High binding, Costar) were coated overnight at rt with 250 ng/well of bacteria or S2 E_ZIKV_, EDI/II_ZIKV_ or EDIII_ZIKV_ protein diluted in 50 μL/well of PBS. After each subsequent step the plates were washed 3 times with PBST. Then, the plates were blocked for 2h at rt with 150 μL of PBST, BSA (1% w/v) and non-fat milk (5% w/v). After this period, 100μL of serially diluted serum from mice immunized or from ZIKV- or DENV-infected participants (1:500) were applied to each well for 2h at rt. Plates were then incubated for 2h with horseradish peroxidase-labeled goat anti-mouse IgG (1:10,000; KPL) or anti-human IgG (1:16,000; KPL). The plates were vigorously washed, and the enzymatic reaction was developed by the addition of 1 mg/mL of o-phenylenediamine (Sigma) diluted in phosphate–citrate buffer, pH 5, containing 0.03% (v/v) hydrogen peroxide. The enzymatic reaction was stopped by the addition of a solution containing 4N H_2_SO_4_. Plates were read at 492 nm (OD_492_) with an ELISA reader (EnSpire Multimode Plate Reader; PerkinElmer). The antibody titer was determined by the highest dilution of serum that presented an OD_492nm_ between 0.1-0.2.

For serum absorption, the ELISA was performed essentially as described above, with an additional step. Serum from DENV^+^ or ZIKV^+^ individuals (1:10) were incubated with 4μg/mL of bacteria E_DENV2_ or EDIII_DENV2_ protein for 1h at rt. Then, the plates coated with bacteria E_ZIKV_, EDI/II_ZIKV_ or EDIII_ZIKV_ protein were incubated for 2 hours with 100μL of adsorbed individual sera (final dilution 1:200).

To compare recognition with the native and denatured proteins, prior to coating, proteins were heated at 100°C for five minutes. Then, the serum from mice immunized were tested against native or denatured proteins.

To evaluate the conformation of E_ZIKV_, EDI/II_ZIKV_ and EDIII_ZIKV_, the assay was performed in a similar way, with some modifications. Plates coated with increasing concentrations (0.625, 1.25, 2.5, 5, 10, 25 or 50μg/mL) of native or denatured recombinant proteins were used for the anti-flavivirus 4G2 (5μg/mL) recognition.

### Plaque reduction neutralization test

A ZIKV isolate from Brazil (ZIKV^BR^), described by Cugola et al. (39), was amplified in Vero E6 cells (ATCC CRL-1586) in complete MEM medium (containing 10% FBS and 1% penicillin/streptomycin (GIBCO)) for 96h. For the neutralization assay, 1x10^5^ Vero CCL-81 cells (ATCC CCL-81) were plated in 24-well plates (Costar) in complete MEM medium and subsequently incubated overnight at 37°C with 5% CO_2_. The following day, serum samples from immunized mice were previously inactivated for 30 minutes at 56°C and incubated in the presence of 100 Plaque Forming Units (PFU) of ZIKV. For this, serum samples were serially diluted (1:10 to 1:320) in 2% MEM medium (containing 2% FBS, 1% penicillin/streptomycin (GIBCO)) and then incubated with 100 PFU of ZIKV per well, for 1h at 37°C with 5% CO_2_. In addition, we included a dose test (DT) – which corresponds to 100 PFU; DT50 (50 PFU), mock (cell only), serum from naive control mice (1:10 to 1:160) and serum from ZIKV-infected participant (positive control, 1:160). Then, plated cells were incubated with the serum-virus mixture for 3h at 37°C. Subsequently, the cells were overlayed with MEM medium with CMC (containing 10% FBS, 1% penicillin/streptomycin, 0.05% Amphotericin B (Fungizone, Gibco) and 1.6% carboxymethylcellulose (CMC, Sigma) and incubated at 37°C. After 4 days of incubation, the medium containing CMC was removed and the wells washed twice with PBS1X. Cells were then fixed with 4% paraformaldehyde (Sigma), stained with 0.2% crystal violet (Sigma) for half an hour and excess dye was removed with distilled water. The percentage of plaque reduction was calculated in comparison to the positive control or DT.

### Splenocyte isolation

Two weeks after the boost, spleens were aseptically removed and splenocytes obtained after red blood cells lysis with ammonium chloride potassium (ACK). Cells were then resuspended in R-10 (RPMI supplemented with 10% of fetal bovine serum, 2 mM L-glutamine, 1% v/v vitamin solution, 1 mM sodium pyruvate, 1% v/v non-essential amino acids solution, 40 μg/mL of gentamicin, 5x10^-5^ M of 2-mercaptoethanol and 1% penicillin/streptomycin (all from Gibco). Cell viability and concentration were estimated using a cell counter (Countess™ Automated Cell Counter, Invitrogen).

### T cell ELISpot assay

IFNγ producing cells were assessed using ELISPOT Mouse IFNg set (BD Biosciences). The procedure was performed according to the manufacturer ‘s instructions. Briefly, 96-well plates (MAIPS 4510, Millipore) were coated with IFNγ capture antibody in PBS and incubated overnight at 4°C. The plates were washed twice and blocked for 2 hours with R10 at rt. Splenocytes were incubated for 18h at 37°C in 5% CO_2_ in the presence of individual ZIKV-peptides (10μg/mL); equimolar amounts of the recombinant proteins E_ZIKV_ (5 μg/mL), EDI/II_ZIKV_ (3.9 μg/mL) or EDIII_ZIKV_ (1.2 μg/mL) produced in bacteria or S2 or R10 (negative control). The plates were washed and incubated with biotinylated anti-mouse IFNγ in PBS plus 10% SFB for 2h at rt. The plates were then washed and incubated with avidin-HRP in PBS plus 10% SFB for 45 minutes at rt in the dark. After extensive washes, the spots were developed with 3-amino-9-ethylcarbazole (AEC) (BD Biosciences) and the number of spots were counted using AID ELISpot Reader System (Autoimmun Diagnostika GmbH, Germany). The number of IFNγ producing cells/10^6^ splenocytes was calculated subtracting unstimulated values from stimulated.

### Analysis of ZIKV-specific T cell cytokine production

To assess ZIKV-specific intracellular cytokine production, splenocytes (1x10^6^ cells/well, in triplicates) were cultured with individual peptides (10μg/mL), equimolar amounts of recombinant proteins E_ZIKV_ (5 μg/mL), EDI/II_ZIKV_ (3.9 μg/mL) or EDIII_ZIKV_ (1.2 μg/mL) produced in bacteria or S2, or R10 (negative control) in the presence of anti-CD28 (2 μg/mL) (BD Pharmigen) and Brefeldin A GolgiPlug™ (BD Pharmigen) for 12h. Then, cells were washed with FACS buffer and surface stained for 30 minutes at 4°C with anti-mouse CD3-APCCy7 (clone 145-2C11), CD4-PerCP (clone RM4-5) and CD8-Pacific Blue (clone 53-6.7). Cells were then fixed and permeabilized using Cytofix/Cytoperm™ kit (BD Pharmigen*)* according to the manufacturer’s instructions. Next, cells were washed with Perm/Wash buffer (BD Pharmigen) and stained with anti- TNFα-PECy7 (clone MP6-XT22) and IFNγ-APC (clone XMG1.2) for 30 minutes at 4°C. Cells were washed twice and resuspended in FACS buffer.

All antibodies were from BD Pharmigen. Samples were acquired on a FACSCanto II flow cytometry (BD Biosciences) and then analyzed using FlowJo software (version 9.9.4, Tree Star). To allow proper compensation, unstained and all single-color controls were performed. The percentage of proliferating cells were calculated by subtracting the values obtained with unpulsed cells.

### Data analysis

The data distribution was analyzed by Saphiro-Wilk normality test. If distribution was normal, we used One or Two-way ANOVA followed by Tukey honestly significantly different (HSD) *post hoc* test. If the data did not pass the normality test, then Kruskal-Wallis followed by Dunn’s multiple comparison test was performed. Statistical analysis and graphical representation were performed using GraphPad Prism version 9.0 software. P values <0.05 were considered significant.

## Results

### Production of the recombinant ZIKV E protein using different heterologous protein expression-systems

Recombinant E_ZIKV_ proteins, including the entire ectodomain (aa 291-690) and the EDI/II_ZIKV_ (aa 291-600) and EDIII_ZIKV_ (aa 601-690) ectodomains, were produced using synthetic genes, containing a consensus sequence derived from different ZIKV isolates, and, subsequently, cloned into prokaryote or eukaryote expression vectors (**Supplementary Figure 1A**). Recombinant proteins expressed either in *E. coli* cells (**Supplementary Figure 1B**) or S2 cells (**Supplementary Figure 1C**) were purified by affinity chromatography and displayed the expected molecular weights, as observed in polyacrylamide gels and Western blots developed with an anti-His tag antibody and with the serum from a ZIKV-infected individual.

### Antigenicity of recombinant ZIKV proteins and cross-reactivity with antibodies raised in DENV-infected patients

**Figure 1:**
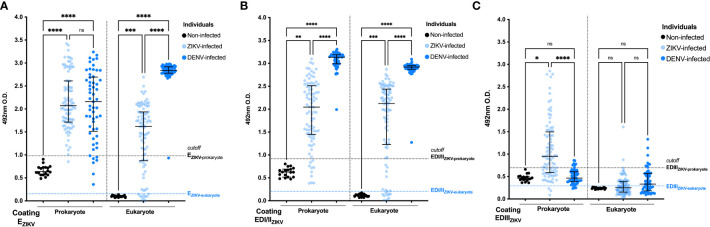
Antigenicity of ZIKV+ and DENV+ sera against recombinant prokaryote and eukaryote EZIKV, EDI/IIZIKV and EDIIIZIKV. Specificity analysis of humoral responses using sera from ZIKV and DENV patients against prokaryote and eukaryote proteins: **(A)** E_ZIKV_, **(B)** EDI/II_ZIKV_ and **(C)** EDIII_ZIKV_. Serum samples from infected individuals (ZIKV^+^ or DENV^+^) are represented in blue circles and samples from negative controls (non-infected) in black. Cutoff: mean O.D. of serum from negative controls plus 3 standard deviations (represented in dashed lines). Statistical significance was measured by nonparametric test Kruskal-Wallis followed by Dunn’s *post hoc* test for multiple comparisons, *p<0.05, ** p<0.01, ***p< 0.001, ****p<0.0001, ns, not significant.

Initially, the recombinant antigens (three produced in *E. coli* and three produced in *Drosophila* cells) were tested in ELISA, as solid phase-bound antigens, and reacted with sera collected from non-infected individuals (n=18) or ZIKV-infected (n=88) individuals. The results shown in **Figure 1** demonstrate that 97.7%, 86.4% and 42% of the ZIKV^+^ samples reacted with prokaryote -E_ZIKV_ (**Figure 1A**), -EDI/II_ZIKV_ (**Figure 1B**), and -EDIII_ZIKV_ (**Figure 1C**), respectively. While 84.1%, 85.2% and 17% of these samples reacted with the corresponding proteins produced in S2 cells. To evaluate the cross-reactivity of the ZIKV recombinant antigens with antibodies raised in DENV-infected subjects, ELISA plates were prepared with the same recombinant ZIKV-antigens and reacted with sera collected from DENV-infected (40) individuals. We observed that 87.7%, 100% and 0% of the DENV^+^ serum samples reacted with E_ZIKV_ (**Figure 1A**), EDI/II_ZIKV_ (**Figure 1B**), and EDIII_ZIKV_ (**Figure 1C**) produced in prokaryotic cells, respectively. In contrast, 100%, 100% and 38.6% of the samples reacted with the corresponding protein domains produced in S2 cells. None of serum samples collected from non-infected participants (ZIKV^-^DENV^-^) reacted with any of the tested recombinant proteins.

Prokaryotic and eukaryotic E_ZIKV_ reacted with ZIKV^+^ and DENV^+^ serum samples, but proteins produced in S2 cells presented lower specificity when tested with DENV^+^ sera (**Supplementary Table 2**). Cross-reactivities with DENV^+^ sera were particularly high with the EDI/II_ZIKV_ (**Figure 1B**), produced in both expression systems. In contrast, higher specificity could be observed with the EDIII_ZIKV_ produced in bacteria (**Supplementary Table 1**).

In further attempts to improve the performance of ZIKV recombinant antigens in serological tests, ZIKV^+^ and DENV^+^ sera were adsorbed with prokaryote E_DENV2_ or EDIII_DENV2_ (**Supplementary Figure 2**). We observed that adsorption of sera from ZIKV-infected individuals with the EDIII_DENV2_ protein, but not with E_DENV2_, decreased the reactivity against the EDIII_ZIKV_ protein. For DENV-infected individuals, with or without adsorption, there was no impact on the recognition of the EDIII_ZIKV_ protein suggesting that most of individuals in our ZIKV-cohort were previously infected with DENV.

### Immunogenicity and induction of neutralizing antibodies in mice immunized with recombinant ZIKV proteins

**Figure 2:**
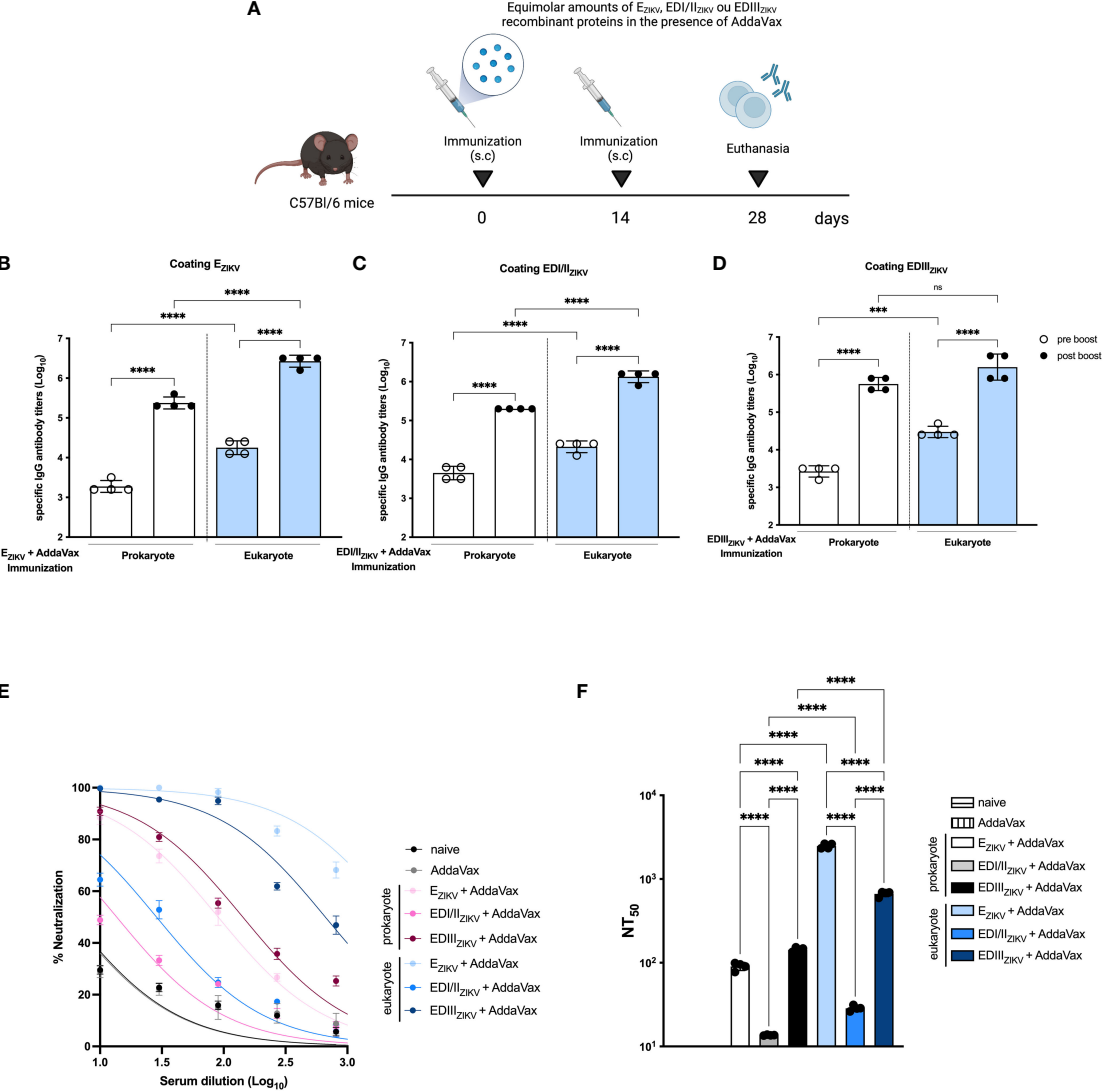
Immunogenicity of ZIKV antigens produced in prokaryotic or eukaryotic cells. **(A)** Immunization strategy (created with Biorender.com). C57Bl/6 mice (n=3 control groups and n=4 experimental groups) received two doses of recombinant antigens (E_ZIKV_, EDI/II_ZIKV_ or EDIII_ZIKV_) in the presence of AddaVax by subcutaneous route. Mice were bled 14 days after each dose to evaluate specific antibody responses by ELISA. Total specific IgG antibody titers on logarithmic scales (Log_10_) on groups that received prokaryote or eukaryote proteins: **(B)** E_ZIKV_, **(C)** EDI/II_ZIKV_ or **(D)** EDIII_ZIKV_. **(E)** Virus neutralization activity of sera collected from immunized mice represented by percentages and **(F)** 50% ZIKV neutralization titers (NT50) of the sera collected from mice after immunization with prokaryote or eukaryote E_ZIKV_, EDI/II_ZIKV_ and EDIII_ZIKV_. Neutralization capacity of naïve or animals that received only adjuvant is also indicated. Statistical significance was measured by One-way ANOVA followed by Tukey’s *post hoc* test, ***p<0.001, ****p<0.0001, ns, not significant. Data represent mean ± SD and are representative of 3 independent experiments.

Next, we evaluated the immunogenicity of E_ZIKV_, EDI/II_ZIKV_ and EDIII_ZIKV_ proteins produced in the two protein expression systems. For that purpose, mice received two doses with equimolar amounts of prokaryote or eukaryote E_ZIKV_, EDI/II_ZIKV_ or EDIII_ZIKV_ administered subcutaneously in presence of the AddaVax adjuvant (**Figure 2A**). Immunization of mice with E_ZIKV_ (**Figure 2B**), EDI/II_ZIKV_ (**Figure 2C**) or EDIII_ZIKV_ (**Figure 2D**), produced either in bacteria or S2 cells, induced antigen-specific antibody responses, particularly after the boosting. Notably, serum IgG titers induced with E_ZIKV_ and EDI/II_ZIKV_ produced in S2 cells were, in general, higher than those achieved in mice immunized with these protein domains produced in BL21 cells. In contrast, IgG titers after immunization with EDIII expressed in both systems were equivalent.

Next, we determined the induction of virus neutralizing antibodies after immunization with the different tested recombinant antigens tested. As shown in **Figures 2E, F** , immunization with E_ZIKV_ and-EDIII_ZIKV_ produced in S2 cells induced higher levels of ZIKV neutralizing antibodies compared to the corresponding antigens produced in bacteria. The PRNT_50_ titers achieved with E_ZIKV_ and EDIII_ZIKV_ produced in S2 cells were 2,482 and 665, respectively (**Figure 2F**). In contrast, immunization with E_ZIKV_ and EDIII_ZIKV_ expressed in bacterial cells induced PRNT_50_ titers of 90 and 143, respectively. As a negative control, mice immunized with the adjuvant alone did not present ZIKV neutralizing antibodies. Next, we assessed the neutralization capacity by passive transfer. Serum of C57Bl/6 mice immunized with the E_ZIKV_ protein was incubated with 100 PFU of ZIKV. After incubation, the serum-virus mixture was administered to AG129 mice (**Figure S3A**). Serum from E_ZIKV-eukaryote_ promotes a slight impact in weight loss (**Figure S3B**), survival rate (**Figure S3C**) and appearance of signs and symptoms (**Figure S3D**) in immunocompromised mice.

### Immunization with E_ZIKV_ expressed in S2 cells delayed the onset of symptoms after challenge with ZIKV

**Figure 3:**
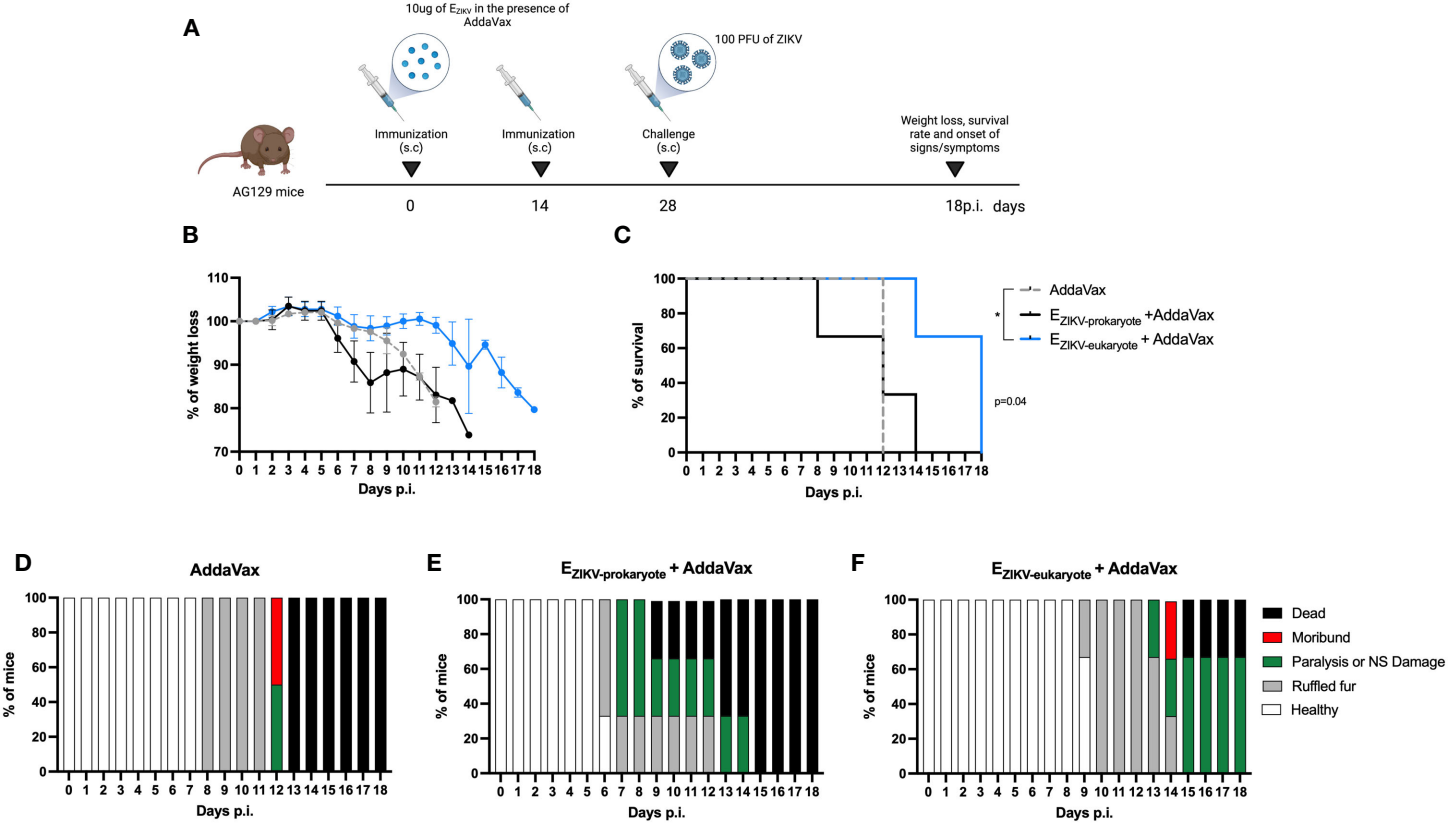
Protection of AG129 mice after immunization with E_ZIKV_ protein expressed in S2 cells. **(A)** Immunization and challenge strategy (created with Biorender.com). AG129 mice (n=2 control groups and n=3 experimental groups) were immunized with two doses of the recombinant protein E_ZIKV_ (10μg, expressed in bacteria or S2 cells) in the presence of AddaVax or adjuvant alone by the subcutaneous route. Fifteen days after the boost, the animals were challenged with 100 PFU of ZIKV into footpads. After challenge, the animals were followed for 15 days to verify **(B)** weight loss, **(C)** survival rate and **(D–F)** signs/symptoms. p<0.05.

To assess whether immunization provide protection, AG129 mice were immunized with two doses of E_ZIKV_ proteins expressed in BL21 or S2 cells. Fifteen days after the boost, mice were challenge with ZIKV (**Figure 3A**). Immunization with E_ZIKV_ protein expressed in S2 cells but not the one expressed in BL21, reduced weight loss (**Figure 3B**), increased survival rate (**Figure 3C**) and delayed the onset of signs and symptoms (**Figures 3D–F**) when compared with the group immunized with AddaVax.

### Antibodies generated after immunization with eukaryotic proteins require conformational epitopes for recognition

**Figure 4:**
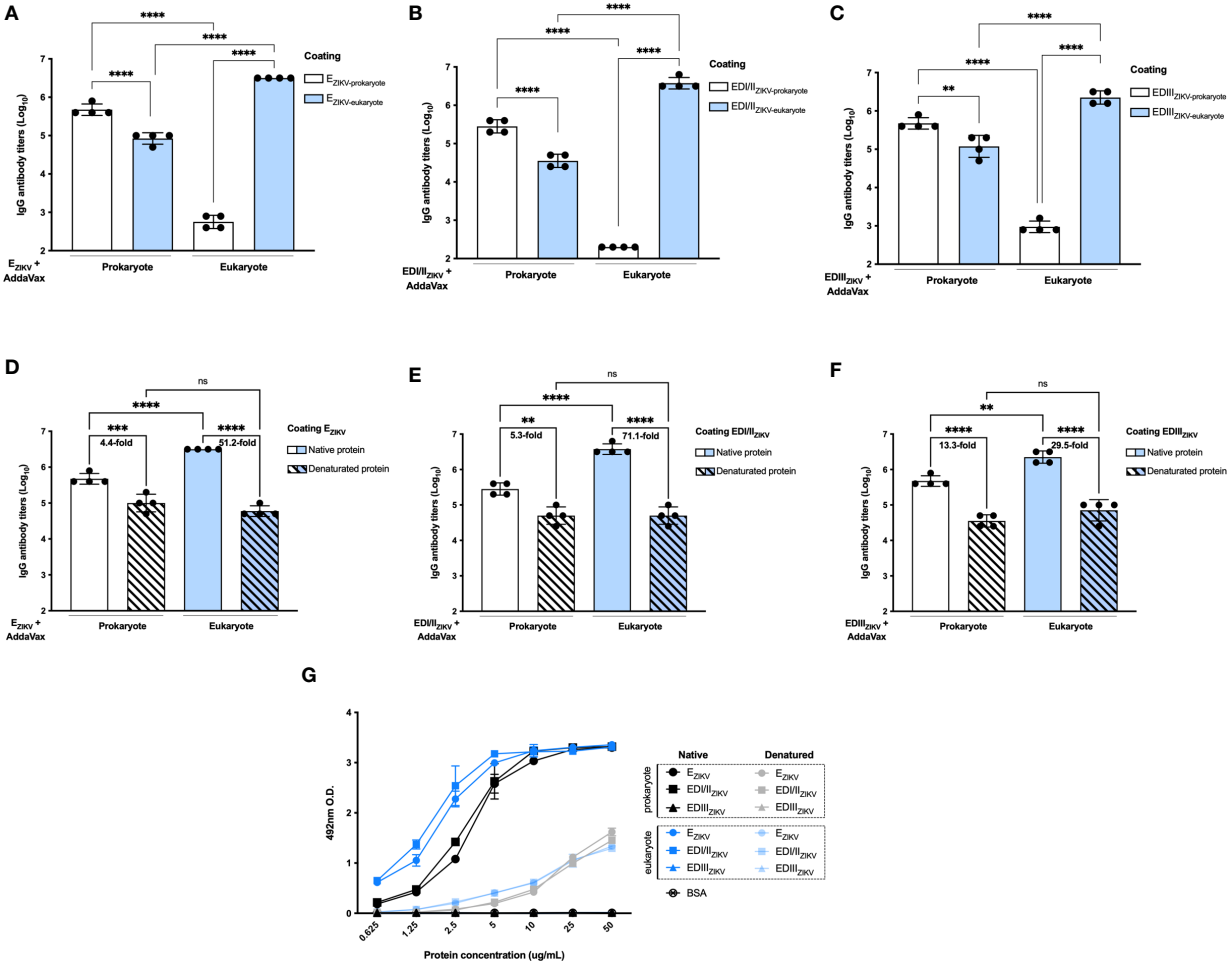
Antigenicity and conformational epitopes of recombinant ZIKV antigens produced in bacterial or insect cells. Binding of serum antibodies from mice immunized with **(A)** E_ZIKV_, **(B)** EDI/II_ZIKV_ and **(C)** EDIII_ZIKV_ expressed in bacteria or insect cells (immunization strategy presented in **Figure 2A**) to recombinant antigens produced in each system. Binding of serum antibodies from mice immunized with **(D)** E_ZIKV_, **(E)** EDI/II_ZIKV_ and **(F)** EDIII_ZIKV_ proteins expressed in bacteria or insect cells (immunization strategy presented in **Figure 2A**) to recombinant antigens produced in each system and submitted or not to denaturation by heat. Numbers on top of bars represent the reduction (in fold change) of the reactivity between intact and denatured proteins in each group. **(G)** Binding of the anti-flavivirus 4G2 monoclonal antibody that recognizes a conformational epitope on the E protein structure. Statistical significance was measured by One-way ANOVA followed by Tukey’s *post hoc* test, **p < 0.01, *** p < 0.001, ****p < 0.0001, ns,not significant. Data represent mean ± SD and are representative of 3 independent experiments.

Antibodies generated after immunization with E_ZIKV_ produced in BL21 recognized this protein generated by S2 cells (**Figure 4A**). In contrast, sera from mice immunized with E_ZIKV_ produced in S2 cells showed reduced reactivity when tested in ELISA coated with this protein produced in BL21 (**Figure 4A**). Similar results were observed with the antibodies raised in mice immunized with recombinant EDI/II_ZIKV_ (**Figure 4B**) or EDIII_ZIKV_ (**Figure 4C**). These data suggest that the antibodies generated in mice immunized with S2-expressed proteins react with conformational epitopes not found in the antigens expressed by bacteria. To further demonstrate such feature, the recombinant proteins were heat denatured before adsorption to ELISA plates. Indeed, antibodies induced in mice after immunization with E_ZIKV_ (**Figure 4D**), EDI/II_ZIKV_ (**Figure 4E**) or EDIII_ZIKV_ (**Figure 4F**), produced either in bacteria or insect cells, displayed reduced reactivity with the corresponding heat-denatured antigens. For E_ZIKV,_ EDI/II_ZIKV_ and EDIII_ZIKV_ produced in bacteria we detected a 4.4, 5.3 and 13.3-fold reduction in antibody reactivity, respectively. For eukaryote-expressed proteins the reduction was 51.2, 71.1- and 29.5-fold, respectively. In addition, binding of the anti-flavivirus 4G2 monoclonal antibody, which recognizes a conformational epitope present in the E protein domain II fusion loop, reacted better with E_ZIKV_ produced in eukaryotic cells compared to the protein generated in bacteria (**Figure 4G**). Heating of the recombinant E_ZIKV_ produced in both expression systems drastically reduced the binding of the 4G2 antibody. Overall, the induction of antibodies that recognize the native E protein and neutralize the virus may require folding that is present only in recombinant ZIKV proteins expressed in insect cells.

### Cellular mediated immunity in mice immunized with recombinant ZIKV proteins

**Figure 5:**
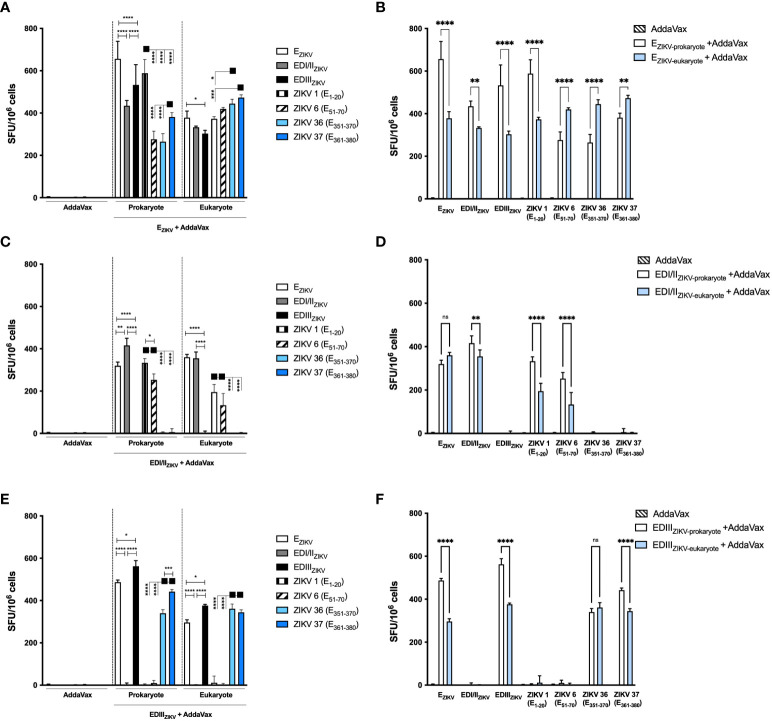
Specific IFNγ-producing cells after immunization with recombinant ZIKV antigens. Analysis of the specific cellular immune responses after immunization of mice with antigens produced in bacterial or insect cells: **(A, B)** E_ZIKV_, **(C, D)** EDI/II_ZIKV_ and **(E, F)** EDIII_ZIKV_. **(B, D, F)** Represents the head-to-head comparation between prokaryote and eukaryote proteins. Fifteen days after the boost the splenocytes were cultured in the presence of equimolar amount of recombinant proteins or ZIKV-specific peptides for 18 hours to evaluate the number of IFN-γ producing cells by ELISpot assay. SFU: spot forming units. Statistical significance was measured by Two-way ANOVA followed by Tukey’s *post hoc* test, *p < 0.05, **p < 0.01, ***p < 0.001, ****p < 0.0001, ns= not significant. Data represent mean ± SD and are representative of 3 independent experiments.

Mice immunized with the recombinant ZIKV proteins were assessed for induction of IFNγ-producing cells in secondary lymphoid organs by ELISpot. The harvested splenocytes were incubated with recombinant proteins and synthetic peptides derived from the E_ZIKV_ (ZIKV 1 (E_1-20_) and ZIKV 6 (E_51-70_)), and EDIII (ZIKV 36 (E_351-370_) and ZIKV 37 (E_361-380_)). Immunization with E_ZIKV_, either produced in bacteria or insect cells, induced IFNγ-producing cells (**Figure 5A**). Mice immunized with E_ZIKV_ produced in *E. coli* induced higher IFNγ-producing cells after stimulation with E_ZIKV_, EDI/II_ZIKV_, EDIII_ZIKV_ and the peptide ZIKV 1(E_1-20_). On the other hand, mice immunized with E_ZIKV_ produced in S2 cells induced IFNγ-producing cells after stimulation with ZIKV 6 (E_51-70_), ZIKV 36 (E_351-370_) and ZIKV 37 (E_361-380_) peptides (**Figure 5B**). In contrast, cells from mice immunized with EDI/II_ZIKV_ or EDIII_ZIKV_ induced specific responses to the corresponding domains. Immunization with the EDI/II_ZIKV_ produced in bacteria or insect cells (**Figure 5C**) induced cellular response against the recombinant E_ZIKV_, EDI/II_ZIKV_, ZIKV 1 (E_1-20_) and ZIKV 6 (E_51-70_) peptides. Immunization with EDI/II_ZIKV_ produced in bacteria induced higher magnitude of cellular responses than the EDI/II_ZIKV_ produced in insect cells for EDI/II_ZIKV_, ZIKV 1 (E_1-20_) and ZIKV 6 (E_51-70_) peptides (**Figure 5D**). Otherwise, immunization with prokaryote or eukaryote EDIII_ZIKV_ (**Figure 5E**) induced cellular responses following stimulation with E_ZIKV_ and EDIII_ZIKV_, as well as, with ZIKV 36 (E_351-370_) and ZIKV 37 (E_361-380_) peptides but, in general, immunization with EDIII_ZIKV_ produced in bacteria induced higher cellular responses than those detected in mice immunized with EDIII_ZIKV_ produced in insect cells, except for peptide ZIKV 36 (E_351-370_) (**Figure 5F**).

**Figure 6:**
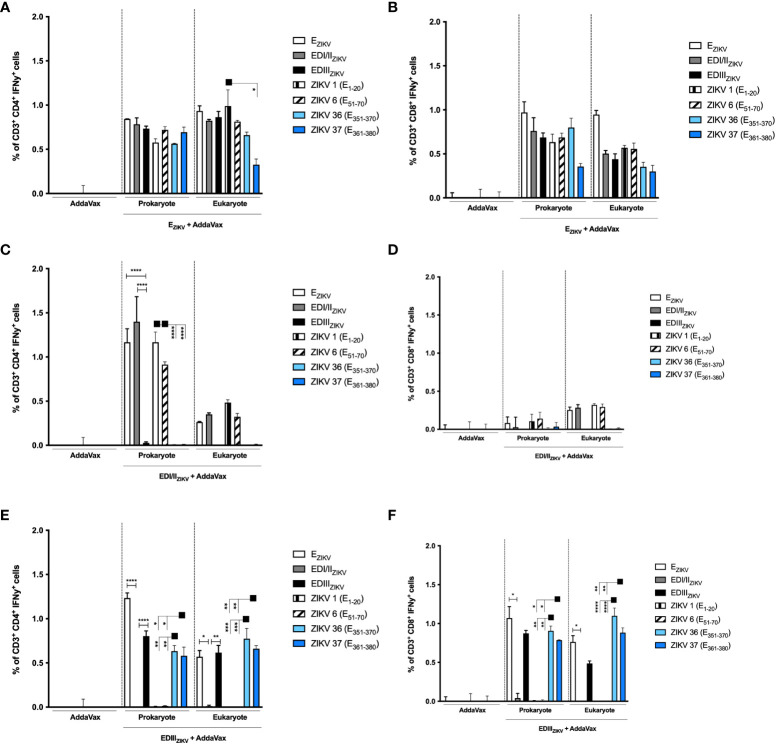
Immunization with recombinant ZIKV proteins induces CD4^+^IFNγ^+^ and CD8^+^ IFNγ^+^ T cell responses. Percentage of **(A, C, E)** CD4^+^IFNγ^+^ and **(B, D, F)** CD8^+^IFNγ^+^ T cells in mice immunized with antigens produced in bacterial or insect cells: **(A, B)** E_ZIKV_, **(C, D)** EDI/II_ZIKV_ and **(E, F)** EDIII_ZIKV_. Fifteen days after the second dose, spleen cells of each mouse were removed and cultured for 12 hours in the presence of equimolar amounts of recombinant proteins or ZIKV-specific peptides, anti-CD28 mAb and brefeldin A. The cells were stained with anti-CD3 and -CD4 antibodies and, subsequently, permeabilized and labeled for intracellular cytokines. The percentage of cells that produce cytokines was calculated by subtracting the values obtained with unstimulated cells. Statistical significance was measured by Two-way ANOVA followed by Tukey’s *post hoc* test, *p < 0.05, **p < 0.01, ***p < 0.001, ****p < 0.0001. Data represent mean ± SD and are representative of 3 independent experiments.

We further analyzed the intracellular cytokine-production by T lymphocytes in mice immunized with the recombinant ZIKV E proteins. Similarly, mice immunized with E_ZIKV_, produced in bacteria or insect cells, activated antigen-specific CD4^+^ IFNγ^+^ (**Figure 6A**) and CD8^+^IFNγ^+^ (**Figure 6B**) T cell responses. Nonetheless, mice immunized with EDI/II_ZIKV_ generated a more potent CD4^+^ T cell responses (**Figure 6C**) to E_ZIKV_, EDI/II_ZIKV,_ ZIKV 1 (E_1-20_) and ZIKV 6 (E_51-70_) peptides than CD8^+^ T cell responses (**Figure 6D**). Mice immunized with EDIII_ZIKV_, either produced in bacteria or insect cells, induced CD4^+^ (**Figure 6E**) and CD8^+^ (**Figure 6F**) T cell responses to E_ZIKV_ and EDIII_ZIKV_ as well as to peptides ZIKV 36 (E_351-370_) and ZIKV 37 (E_361-380_).

In general, a similar profile was observed in TNFα production (**Supplementary Figure 4**) by CD4^+^ and CD8^+^ T cells after immunization with the different ZIKV E proteins. Immunization with prokaryote and eukaryote E_ZIKV_ expressed proteins induced CD4^+^TNFα^+^ (**Supplementary Figure 4A**) and CD8^+^TNFα^+^ (**Supplementary Figure 4B**) T cells for all stimuli. On the other hand, immunization with the EDI/II_ZIKV_ and EDIII_ZIKV_ domains of prokaryotes or eukaryotes induced CD4^+^TNFα^+^ (**Supplementary Figure 4C and 4E**, respectively) and CD8^+^TNFα^+^ (**Supplementary Figure 4D and 4F**, respectively) T cells against E_ZIKV_ protein, the same domain used in immunization and also against correlated peptides: EDI/II region: ZIKV 1 (E_1-20_) and ZIKV 6 (E_51-70_) peptides; EDIII region: ZIKV 36 (E_351-370_) and ZIKV 37 (E_361-380_) peptides. Collectively, these results demonstrated that ZIKV recombinant antigens, produced either in bacterial or insect cells, present similar capacities to stimulate T cell-dependent responses.

## Discussion

The spread of ZIKV infection worldwide has highlighted the need for specific and sensitive diagnostic tools, as well as the development of effective vaccine formulations. ZIKV is endemic in tropical regions of the world and follows a distribution pattern similar to that of DENV. Serological tests are key for adequate diagnostic and epidemiological studies of arboviruses. The specificity of current diagnostic tests is compromised by similarities shared between these viruses (41–43). The ZIKV and DENV E proteins share 54 to 57.8% sequence identity on the protein sequence, including each of the three functional domains, with conserved linear and structural epitopes. These features contribute to cross-reactivity of polyclonal antibodies raised in individuals infected with these viruses, which poses a great challenge for the development of sensitive and specific serological diagnostic tests (12). It is well established that antibodies that develop in response to infection by one flavivirus may also recognize others (44). The Zika CDC MAC-ELISA and InBios assays show low specificity (45) due to the homology present in the envelope region between DENV and ZIKV, while other tests present lower sensitivity (45–47). The similarity between the envelope proteins of the different flaviviruses highlighted the importance of developing recombinant proteins with appropriate conformation, activity, and functionality (29). On the other hand, the use of recombinant forms of structural proteins also represents a relevant strategy for the development of anti-DENV and anti-ZIKV vaccines but questions regarding the immunological differences of antigens, particular those derived from the E protein, produced in bacterial or eukaryotic cells are still open (29, 30, 48–52). Here we studied the features of E_ZIKV_, EDI/II_ZIKV_ and EDIII_ZIKV_ produced in two different expression systems. We observed that the prokaryote-expressed EDIII_ZIKV_ protein, instead of the one produced in eukaryotic cells, presented the best combination of sensitivity and specificity to distinguish between ZIKV^+^ and DENV^+^ participants. We believe that when ZIKV EDIII is produced in insect cells, but not in bacteria, some epitopes could be hidden or not correctly folded, limiting its recognition by antibodies present in sera of infected individuals. In fact, the recombinant EDIII_ZIKV_, as a domain of the ZIKV envelope protein, presents large surface areas hidden in the dimerized E protein that, when expressed individually as a single domain, become exposed (53). In fact, serologic reactivity against EDIII_ZIKV_ produced in the HEK293-6E cell system has been shown to vary greatly between individuals infected with ZIKV, with most of the participants having low titers (15). On the other hand, a recent study demonstrated that the EDIII_ZIKV_ protein produced in bacteria showed 90% specificity and 92% sensitivity to detect Zika infected patients, providing a good basis for a diagnostic assay (54).

The recent ZIKV outbreak also highlighted the urgency of an effective vaccine. Modern approaches evaluated the immunogenicity and protection of subunit vaccines encoding E and prM-E proteins in both preclinical and phase I and II clinical trials (23). Viral vectored and nucleic acid vaccines (DNA and mRNA) encoding prM-E induced potent neutralizing antibodies, T-cell responses, and protection in animal models (48, 49, 55–57). Here, we compare the immunogenicity of different subunit vaccines based on the ZIKV envelope region in a preclinical setting. Although all eukaryote and prokaryote proteins induced high specific antibody titers in mice, we observed a difference in their ability to neutralize ZIKV. Immunization with EDI/II_ZIKV_ expressed in both systems was unable to induce neutralizing antibodies. In fact, Stettler et al (14) demonstrated that antibodies against EDI/II_ZIKV_ derived from convalescent ZIKV individuals were weakly neutralizing and cross-reactive, leading to increased DENV infection in mice. Even though EDI/II induces antibodies with lower neutralization capacity, this region may still contribute to the induction of helper T cells, that, in turn, may help B cells to produce neutralizing antibodies (50). Previous studies already demonstrated that EDIII is the main target of neutralizing antibodies (14, 15, 27). Immunization with this domain induces potent neutralizing antibodies (30, 31, 58, 59). Here, animals that received E_ZIKV_ and EDIII_ZIKV_ expressed in S2 cells showed higher neutralizing antibody titers than mice immunized with bacteria-expressed proteins. Furthermore, AG129 mice immunized with the E_ZIKV_ protein expressed in S2 cells or passively transferred with anti-E_ZIKV_ sera presented delayed disease progression after challenge with ZIKV. Corroborating our results, Qu et al. also showed that EDIII_ZIKV_ expressed in insect cells induced neutralizing antibodies in mice and the passive transfer of sera conferred protection (59). In addition, the administration of E-specific mAbs was also able to reduce viral infection (24). However, other research groups obtained contrasting results. For example, while truncated prokaryote-expressed E_ZIKV_ (30) or EDIII_ZIKV_ (31) were shown to induce high titers of neutralizing antibodies, no difference in immunogenicity and protection were obtained when a version of the E protein (E80) was produced in eukaryotic and prokaryotic systems (40).

CD4^+^ T cells is essential for the protective immunity against ZIKV, since their depletion reduced the generation of anti-ZIKV antibodies (50, 60) and CD8^+^ T cell responses (61). Furthermore, CD8^+^ T cells are important during flavivirus infections as they contribute to protection in B cell-deficient mice (51), and their lack increases mortality in ZIKV-infected mice (62). In a vaccination context, cellular immune responses may also play an important role in the generation of a long-lasting immunity. The results of a phase 1 clinical trial of a ZIKV inactivated vaccine demonstrated that neutralizing antibody response declined significantly by week 16 (52). The sharp drop in neutralizing antibody titers may be related to the poor ability of the inactivated vaccine to induce cellular immune responses, particularly the lack of CD8^+^ T cells (50). Here, immunization with all ZIKV envelope proteins induced specific IFNγ-producing cells as well as CD4^+^ and CD8^+^ T cells able to produce IFNγ or TNFα, in a similar way. Similar results were obtained previously in a phase I clinical trial with a DNA-based ZIKV vaccine (56) and reinforce the perspective for the development and use of subunit vaccines against ZIKV as well as other flavivirus infections.

In summary, the present study evaluated the properties of different domains of the ZIKV E protein (E_ZIKV_, EDI/II_ZIKV_ and EDIII_ZIKV_) produced in two different heterologous expression systems. The results demonstrated that EDIII_ZIKV_ produced in bacterial cells retained better antigenicity than the corresponding protein produced in insect cells, considering both sensitivity and, particularly, specificity to identify ZIKV response in patients from DENV endemic regions. On the other hand, E_ZIKV_ and EDIII_ZIKV_ produced in insect S2 cells showed a better performance as vaccine antigens due to their ability to induce neutralizing antibodies in vaccinated mice. Nonetheless, antigens produced in both expression systems showed similar immunological activities regarding activation of antigen-specific T cell responses. The experimental data provided here could be used as a basis for the rational design of serological tests and may contribute to the refinement of subunit vaccine candidates against ZIKV.

## Data availability statement

The original contributions presented in the study are included in the article/**Supplementary Material**. Further inquiries can be directed to the corresponding author.

## Ethics statement

The studies involving human participants were reviewed and approved by the Ethics Committee (protocol CAAE 68688117.0.0000.5505). The patients/participants provided their written informed consent to participate in this study. The animal study was reviewed and approved by UNIFESP Institutional Animal Care and Use Committee (IACUC) -protocol number #2020100418.

## Author contributions

VL: Investigation, Methodology, Formal analysis, Validation, Writing - original draft, Visualization. BA and JA: Investigation, Methodology. TR, MY, SP, MB and LP: Investigation. KC: Resources. RS: Resources, Writing – review & editing. LF: Methodology, Resources, Writing – review & editing. SB: Conceptualization, Methodology, Resources, Writing - review & editing. DR: Conceptualization, Methodology, Resources, Writing - original draft, Writing - review & editing, Visualization, Supervision, Project administration, Funding acquisition. All authors contributed to the article and approved the submitted version.
