# Supplementary material for: Diagnostic and vaccine potential of Zika virus envelope protein (E) derivates produced in bacterial and insect cells
Source: Front Immunol. 2023 Mar 16;14:1071041. doi: 10.3389/fimmu.2023.1071041 (PMC10060818; doi:10.3389/fimmu.2023.1071041)
Supplement: Supplementary file 1 [file DataSheet_1.docx]

**Supplementary Material**

**Figure S1: Expression, purification, and characterization of ZIKV recombinant antigens (E_ZIKV_, EDI/II_ZIKV_ and EDIII_ZIKV_) produced in *E. coli* and S2 cells.** **(A)** Schematic representation of the amino acid sequences of the recombinant ZIKV-envelope proteins. Electrophoretic analyses of recombinant antigens produced in **(B)** bacterial (prokaryote) and **(C)** *Drosophila* (eukaryote) cells. The E_ZIKV_, EDI/II_ZIKV_ and EDIII_ZIKV_ recombinant antigens were purified by affinity chromatography, sorted in polyacrylamide gels and, subsequently, submitted to Western blots developed with anti-His tag antibodies and serum collected from a ZIKV^+^ participant. Molecular weight in kDa.


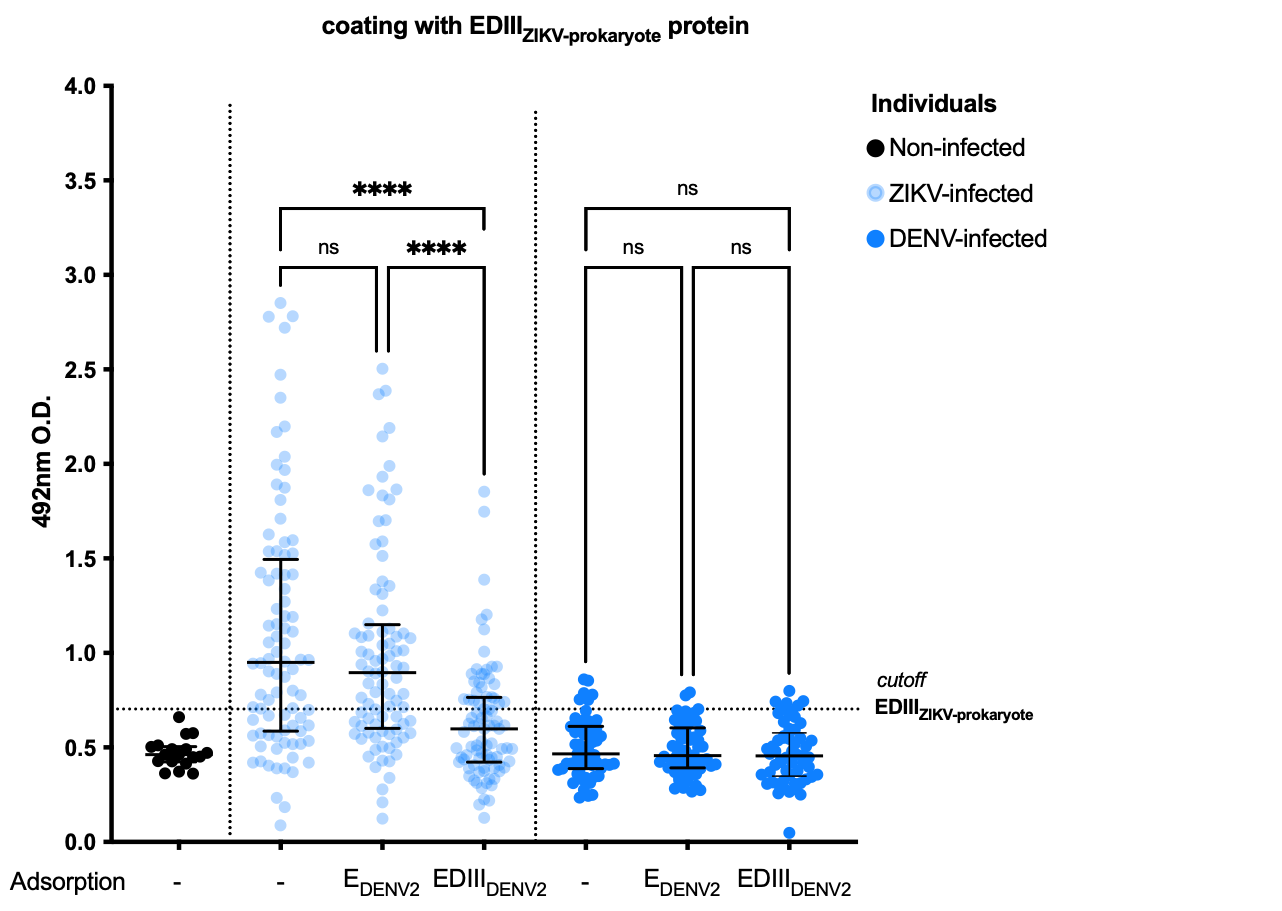


**Figure S2: Analysis of ZIKV^+^ and DENV^+^ serum antigenicity against prokaryote EDIII_ZIKV_ after adsorption with DENV2-recombinant proteins.** Analysis of humoral responses using sera from ZIKV and DENV patients against prokaryote EDIII_ZIKV_, with or without adsorption using recombinant E_DENV2_ or EDIII_DENV2_. Samples from infected individuals (ZIKV^+^ or DENV^+^) are represented in blue circles and samples from negative controls (non-infected) in black. Cutoff: mean O.D. of serum from negative controls plus 3 standard deviations (represented in dashed lines). Statistical significance was measured by nonparametric test Kruskal-Wallis followed by Dunn's post hoc test for multiple comparisons. ****p<0.0001, ns= not significant.


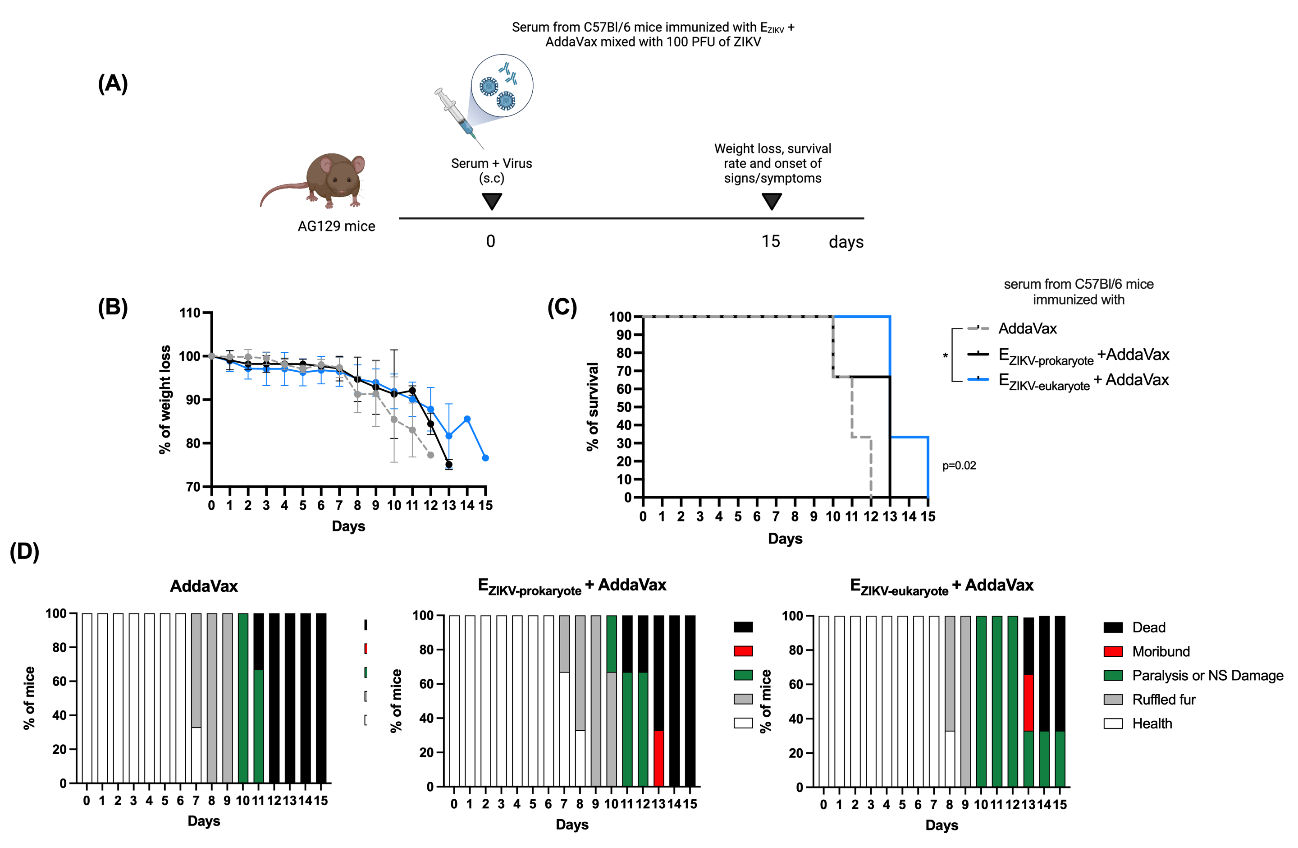


**Figure S3: *In vivo* neutralization.** **(A)** *In vivo* neutralization strategy (created with Biorender.com). Serum from C57Bl/6 mice (n=3 control groups and n=3 experimental groups) immunized with the recombinant protein E_ZIKV_ (10μg, expressed in bacteria or S2 cells) in the presence of AddaVax or the adjuvant alone was incubated with 100 PFU of ZIKV for 1 hour. After this period, AG129 mice received the serum-virus mixture into footpads and were followed for 15 days to verify **(B)** weight loss, **(C)** survival rate and **(D)** signs/symptoms.


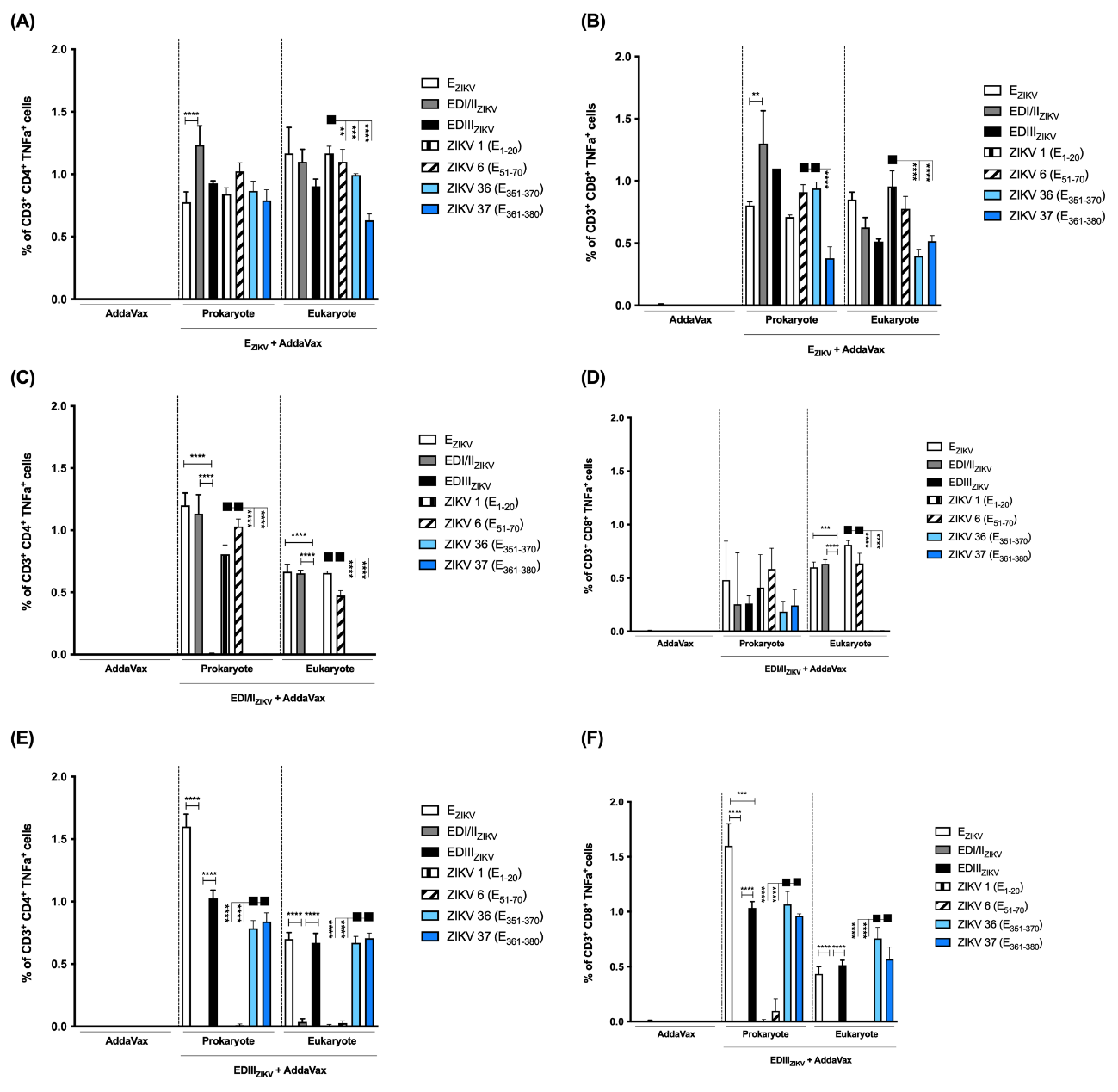


**Figure S4: Immunization with recombinant ZIKV envelope proteins induces CD4^+^TNFα^+^ and CD8^+^TNFα^+^ T cells.** Percentage of **(A, C, E)** CD4^+^TNFα^+^ and **(B, D, F)** CD8^+^TNFα^+^ T cell after immunization of mice with prokaryote and eukaryote **(A, B)** E_ZIKV_, **(C, D)** EDI/II_ZIKV_ and **(E, F)** EDIII_ZIKV_. Fifteen after the second dose, the spleen of each animal was removed and cultured for 12 hours in the presence of equimolar amounts of recombinant proteins or ZIKV-peptides, anti-CD28 and brefeldin A. The cells were stained with anti-CD3, -CD4 and -CD8, then permeabilized and labeled for intracellular cytokines. After determining the populations of T cells that produce cytokines by flow cytometry. The percentage of cells that produce cytokines was calculated by subtracting the values ​​from the unstimulated cell culture. Statistical significance was measured by Two-way ANOVA followed by Tukey’s post hoc test, **p<0.01, ***p<0.001, ****p<0.0001. Data represent mean ± SD and are representative of 3 independent experiments.

**Table 1: Primers**

| Bacteria | Forward | Reverse |
| --- | --- | --- |
| EDI/II_ZIKV_  ectodomain | 5’-GGGCTAGCATTCGTTGCATCG -3’ | 5’-CCCTCGAGCGCGGTGCACAGGCTGTA-3’ |
| EDIII_ZIKV_ ectodomain | 5’-GGGCTAGCGCGTTCACCTTTACCAAAATT-3’ | 5’-GGCTCGAGCCAGTGGTGGGT-3’ |
| S2 Drosophila | **Forward** | **Reverse** |
| E_ZIKV_ | 5’-GGCCATGGATCAGGTGTATCGGGGTCAG-3’ | 5’-GGACTCGAGCGCCAATGATGGGTAATCTTCTTC-3’ |
| EDI/II_ZIKV_  ectodomain | 5’-GGCCATGGATCAGGTGTATCGGGGTCAG-3’ | 5’-CCACTCGAGCGGGCTGTGCACAGGGAGTAG-3’ |
| EDIII_ZIKV_ ectodomain | 5’-GGCCATGGGCCTTCACCTTCACCAAGAT-3’ | 5’-GGACTCGAGCGCCAATGATGGGTAATCTTCTTC-3’ |

**Table 2: Percentage of sensibility, specificity, PPV and NPV of E_ZIKV_, EDI/II_ZIKV_ and EDIII_ZIKV_ proteins**

|  | **Sensitivity** | | | **Specificity** | | |
| --- | --- | --- | --- | --- | --- | --- |
|  | **E_ZIKV_** | **EDI/II_ZIKV_** | **EDIII_ZIKV_** | **E_ZIKV_** | **EDI/II_ZIKV_** | **EDIII_ZIKV_** |
| **Bacteria** | 97.73 | 86.36 | 42.05 | 12.28 | 0 | 100 |
| **Drosophila** | 84.09 | 85.23 | 17.05 | 0 | 0 | 61.4 |

|  | **PPV** | | | **NPV** | | |
| --- | --- | --- | --- | --- | --- | --- |
|  | **E_ZIKV_** | **EDI/II_ZIKV_** | **EDIII_ZIKV_** | **E_ZIKV_** | **EDI/II_ZIKV_** | **EDIII_ZIKV_** |
| **Bacteria** | 63.24 | 57.14 | 100 | 77.78 | 0 | 52.78 |
| **Drosophila** | 56.49 | 56.82 | 40.54 | 0 | 0 | 32.41 |

***PPV: positive predictive value; NPV: negative predictive value**
